# Distinguishing Representations as Origin and Representations as Input: Roles for Individual Neurons

**DOI:** 10.3389/fpsyg.2016.01537

**Published:** 2016-09-30

**Authors:** Jonathan C. W. Edwards

**Affiliations:** University College LondonLondon, UK

**Keywords:** mental representation, percept, grandmother cell, pontifical cell, gnostic cell

## Abstract

It is widely perceived that there is a problem in giving a naturalistic account of mental representation that deals adequately with the issue of meaning, interpretation, or significance (semantic content). It is suggested here that this problem may arise partly from the conflation of two vernacular senses of representation: representation-as-origin and representation-as-input. The flash of a neon sign may in one sense represent a popular drink, but to function as a representation it must provide an input to a ‘consumer’ in the street. The arguments presented draw on two principles – the neuron doctrine and the need for a venue for ‘presentation’ or ‘reception’ of a representation at a specified site, consistent with the locality principle. It is also argued that domains of representation cannot be defined by signal traffic, since they can be expected to include ‘null’ elements based on non-firing cells. In this analysis, mental representations-as-origin are distributed patterns of cell firing. Each firing cell is given semantic value in its own right – some form of atomic propositional significance – since different axonal branches may contribute to integration with different populations of signals at different downstream sites. Representations-as-input are patterns of local co-arrival of signals in the form of synaptic potentials in dendrites. Meaning then draws on the relationships between active and null inputs, forming ‘scenarios’ comprising a molecular combination of ‘premises’ from which a new output with atomic propositional significance is generated. In both types of representation, meaning, interpretation or significance pivots on events in an individual cell. (This analysis only applies to ‘occurrent’ representations based on current neural activity.) The concept of representations-as-input emphasizes the need for an internal ‘consumer’ of a representation and the dependence of meaning on the co-relationships involved in an input interaction between signals and consumer. The acceptance of this necessity provides a basis for resolving the problem that representations appear both as distributed (representation-as-origin) and as local (representation-as-input). The key implications are that representations in the brain are massively multiple both in series and in parallel, and that individual cells play specific semantic roles. These roles are discussed in relation to traditional concepts of ‘gnostic’ cell types.

## Introduction

Concepts of mental representation are widely invoked in neurobiology, linguistics, artificial intelligence, and philosophy. Yet, as Seager and Bourget (2007) note: “there is no acknowledged theory of mental representation.” This appears to be partly because people differ in terms of the explanatory work they want such a theory to do (Stich, 1992). It also reflects an impasse in reaching a consensus on how mental representations could fit into a naturalistic account of the brain; what sort of substrate, or causal nexus could support a mental representation, and how? I shall argue that these are interdependent questions and that a careful assessment of the logical constraints on substrate, in terms of physical dynamics and their location, may clarify the ways in which mental representation may be a useful concept, as well as vice versa.

From the outset I wish to emphasize that the problem I address relates only to what may be called ‘occurrent’ or ‘active’ representations in which signals are sent and received on specific occasions. There is another use of the term that might be called a ‘dispositional representation’ – an acquired pattern of cellular connectivity underlying memory, knowledge, or concept acquisition, that disposes the brain to generate occurrent representations in response to stimuli (Simmons and Barsalou, 2003). I will be using ‘representation’ to mean ‘occurrent representation.’

The naturalization problem is not so much about whether a representation is to the right, left, front or back of the brain, or what connection tracts are involved. The more basic problem is defining the *type*, or level, of biophysical location that could support a fitting causal role, and with appropriate information capacity (‘bandwidth’). There are those who would argue that we have a rough answer: that representations can be equated with patterns of neural activity, or firing. However, as discussed below, this fails to address key problems, justifiably of concern to philosophers of mind. Meaning is *not* to be solved so easily.

It might be argued that searching for a detailed substrate type for mental representation is overly reductionist or, in theoretical modeling terms, simply premature. It might even be considered immaterial to understanding of how a representation can have a meaning, either in terms of external referents or internal ‘meaning to the subject.’ However, I think the search is justified on the following grounds. Firstly, spatial pattern is about the only way meaning can be encoded in a brain at any point in time, as far as we know, so at least *type* of spatial pattern and location is likely to be central to a theory of meaning. Secondly, recognizing that reductive analysis of mechanism is only part of the story does not mean that fruitful progress in neural mechanisms should be abandoned half-finished and replaced by hand-waving. Rather than, as Marr (1982) advocated, treating the biophysical and ‘functional’ levels of analysis as incommensurable, to be able to test viability of theories I believe, with Trehub (1991), that we need some idea of how and where they could correspond.

Moreover, the ability to suggest at least one plausible physical example for any theoretical model is a requirement that is arguably never premature. A search for such examples can render explicit contradictions in popular concepts. The key proposal here is that neuropsychology may benefit from a greater focus on the input aspect of mental representation. The author’s background is in immunology. It was not until we insisted on a grounding in a dynamics of integration of signals into individual cells that we began to understand leucocyte behavior in immune recognition and memory (Male et al., 2012). Hypotheses that could not be so grounded were discarded. The gap between work on post-synaptic integration (e.g., Branco and Häusser, 2011; Smith et al., 2013; Ishikawa et al., 2015) and psychology may still be harder to bridge but the possibility of grounding in plausible input mechanisms should be an acid test of all models of mental representation.

## The Nature of Mental Representations

Representation is a term used in a variety of ways that are not always transparent. It is not simply ‘re-presentation,’ and not just because ‘presentation’ might be a better label. It can also imply ‘proxy’ or ‘symbol.’ In the mental case, where representations do not resemble their referents in any simple way, the meaning of the term will be preconditioned not only by presumptions about how brains work but also metaphysical standpoint. A materialist may think in terms of brain states representing external ‘things’ whereas someone taking a dynamist or structural realist approach (as I do) may think in terms of internal dynamic relations representing external dynamic relations (Ladyman and Ross, 2007). There will also be different views on how these concepts relate to subjectivity or phenomenality. To clarify the way ‘representation’ relates to meaning it may help to consider two main purposes to which the term ‘mental representation’ is put.

Mental representation may be invoked simply as part of an account of the human brain as a machine that generates outputs from inputs. A mental representation can be seen as the equivalent of local currents or magnetizations in a computer. As long as we accept that brain cells send messages around in a way vaguely similar to computer components, we can consider the nature of mental representations in this context as just a technical issue, like the difference between Microsoft Windows and Mac OS-X, without raising too many philosophical questions. ‘Representation’ is being used here purely to imply some internal dynamics that co-vary usefully with external world dynamics.

There is, nevertheless, even here, a need to define a representation more precisely than just that total pattern of brain activity that arises in a specific context, whether the presence of a red square or blue circle, or when thinking ‘I suspect the recession will double-dip.’ A representation is not just a pattern of events; it is a pattern with a causal role. A red square will trigger patterns in the retinae, geniculate bodies, primary and secondary visual cortices, temporal, parietal and frontal lobes, all with different causal roles. To function, the content of any individual representation must be available to some functional component at a causal nexus: what Millikan calls a ‘consumer’ (Ryder et al., 2012). Thus we may need to talk of many mental representations at many levels rather than a single representation. That then begs the question of which mental representations are those envisaged by philosophers and linguists such as Fodor (1985) or Dretske (1986) and what their consumers are.

The second motivation for talking about mental representations is in the context of questions about first person experience, as conceived from positions on the nature of ‘mentality’ ranging from Cartesian to eliminitivist (Stich, 1992). Thus ‘mental representation’ is often used to imply an associated experience, in which operational meaning is somehow ‘interpreted.’ This may be as a ‘percept,’ as when something is viewed or heard, or a ‘mental image,’ as when retrieving memories, thinking of a scene or sound, or in dreams (Fodor, 1975; Kosslyn, 1994).

There is a general assumption that there is only *one instance* of this ‘percept’ type of representation in a brain at a time, and there has been extended debate over whether this is local or distributed (e.g., Barlow, 1972; Fodor and Pylyshyn, 1988; Marcus, 2001), which remains unresolved. It is suggested here that this may reflect confusion about what we should expect the biophysical processes underlying a representation, of the ‘percept’ type, to consist of and where they might be – and that the assumption that there is only one such representation needs challenging.

There are those who, probably rightly, point out that a first person account of mental representation will ultimately be redundant to a description of its physical dynamics (e.g., Churchland, 1992). The mistake, I believe, is to take this as a reason for discounting the first person account. Even granted that representations of the percept type may form a tiny minority of the total, and quite apart from the desire to know how there comes to be a first person account, it is likely that without heuristic clues from experience and the language we use to describe it the causal dynamics of all our representations will remain intractable. However tidy it may feel to regard talk of ‘phenomenality’ as outside physical science, I follow those who argue that there is a strong case for accepting that ‘phenomenal experience’ plays a crucial role in all science, as the medium of observation, and that we should be happy to make all use of it we can. Thus, mental representations associated with experience or ‘feel,’ whether percepts or ‘current belief states’ (Crane, 2014) are not only those of greatest philosophical interest but may also be particularly worth exploring for their potential to shed light on mental processes in general. I shall therefore focus on such representations from now on, taking sensory percepts as the paradigm.

## General Causal Principles

Unless there are good reasons otherwise, an account of a representation-as-percept in a brain should follow causal principles used elsewhere in physical science, where possible confirmed by experimental neurophysiology. Two such principles are particularly relevant. The first is the neuron doctrine. The second is that the content of a percept will be encoded in signals that form inputs to some physical domain.

The neuron doctrine, in essence, is the principle that brain function (*qua* ‘thinking’) can be explained by the interactions of separate neuronal units (Gold and Stoljar, 1999). Each neuron is a discrete computational (in the broad sense of having rule-based input–output relations) unit, conforming to biophysical laws. The timing of firing of a neuron is determined by chemical and electrical interactions between the cell and its immediate environment. All cause and effect relations occur locally. The neuron doctrine does not preclude other levels of explanation in terms of groups of cells or macroscopic brain domains, but holds that these can be broken down, without residue, to an account of individual cell interactions.

Some have suggested that the neuron doctrine should be replaced by a description of brain function at a ‘global’ level (Gold and Stoljar, 1999). However, since the causal biophysical pathways of the neuron doctrine are not seriously in doubt it is unclear that a global description can be an alternative, rather than just a higher-level analysis grounded in the same local dynamics. There may be a temptation to suggest that some of the perplexing aspects of mental representation can only be accounted for using approaches such as systems theory or non-linear dynamics that might be seen to give an ‘emergent’ dynamic ‘greater than the sum of the parts.’ However, without clear evidence it seems safer to assume that, as Barlow (1994) says, all causal relations pass through the bottlenecks of individual neurons.

The second premise is as fundamental but less often articulated. It underlies Rosenberg’s (2004) concept of receptivity and Millikan’s idea of ‘consumer’ and is laid out in explicit neurological terms by Orpwood (2007). The representations we call percepts must be based on the co-availability of certain signals to some neuron-based domain, i.e., they must be inputs to such a domain, which will also generate outputs in response that allow the percept to be ‘reported.’ (Reporting may be a complex indirect process but the basic point is unaffected.) Something has to receive the signals that encode a percept, whether these are derived originally from sense organs or other sources as in dreams. An un-received signal does not even qualify as a signal, since reception is entailed in the concept.

This might seem self-evident. However, this second premise is worth emphasizing because literature on consciousness often appears to take a different view. Representations may be seen as associated with computational or ‘information processing’ operations, which involve not inputs but input–output relations, or ‘roles in the world’ – the essence of ‘functionalism’ (Fodor, 1975; Block, 1996). The ‘content’ of the representation is then seen as being dependent not only on the effect of the world on the computational unit but also on the effect of the unit on the world. This appears to imply that if percepts belong to physical domains then those domains are in some way *acquainted with, or informed by, their outputs* (effects on the world) as well as their inputs. This is self-contradictory for any computational system that obeys standard concepts of causality – what something has access to *is* its input – and neuroscience consistently indicates that these concepts of causality hold good.

I must emphasize that this is a low-level analysis dealing with individual neuro-computational steps. Events within feedback systems taken as whole, as in anticipatory models of perception (Hommel, 2009) can, in a broader sense, be considered as representing a certain action/perception scenario but even here it is not the input/output relation that gives the content, but the particular pattern of signals (‘the data’), considered either as cellular outputs or inputs.

Both in neuroscience and philosophy, representations are often considered in terms of patterns of cell activity, with no specific reference to input or output. The problem here is that to consider a pattern as an operant representation implies that the total activity pattern is accessible to something. A pattern of activity of 73,456 out of a bank of 1,000,000 right occipital cells might seem to represent a scene. However, each of these cells may have 10,000 branches to its axonal output, some feeding forward, some back. Only 6,228 cells may send branches to each of a bank of temporal cells, and 18,992 to a bank of prefrontal cells (through any one direct or indirect route) and, moreover, there will be variation (and plasticity) in this between individual sending and receiving cells in each bank. Although the activity of the 73,456 cells is a representation in a certain legitimate vernacular sense, there seems to be another important sense in which it underpins, together with whatever other ‘null cells’ whose non-firing may contribute critically to the content being conveyed, not one, but many, representations-as-inputs, diverse in content and function.

In other words, an act of representation must ultimately imply an input *to* something specified. It is sometimes implied that there are no ‘inner receiving entities’ for representations in a brain, but, again, this is inconsistent with our understanding of causality. To be part of a causal chain, and thus reportable, the information encoded in a representation must be made available to something that generates a response. A word of text in a forgotten language embedded in an opaque medium that cannot be removed without destroying the text cannot function as a representation. Similarly, a pattern of lines of cellular activity in my visual cortex that bears a homotopic relation to a pattern of tree trunks I am viewing is not acting as a spatial representation *by dint of homotopy*, since no part of me, including the cells themselves, is informed of the spatial relations of active and inactive cells. The cells provide a representation in the form of presenting sensory data to other parts of my brain through patterns of downstream synaptic transmission, but the homotopic spatial relation of their cell bodies is itself of no consequence. Representation must be linked to a causal path.

Inner receiving entities are often rejected as ‘homuncular’ and criticized on grounds that shifting the problem of the input/percept relationship for a brain to a subdomain of brain leaves the problem unchanged and therefore invokes infinite regress. The implication of regress is, however, *non sequitur*. If the problem is the same as for the whole brain then that must surely also suffer from the regress. The reverse conclusion applies: if the problem has *any* solution for the brain it may *also* have a solution for a homuncular subdomain and it may only have a solution there (see also Fodor, 1975, p. 189) Thus, even Dennett’s (1988) homunculi that ‘repeat entirely the talents they are rung in to explain’ are only straw bogeymen. Homunculi are in fact usefully rung in to deal with practical computational issues.

There is no doubt that treating representations as inputs to specific neural structures raises difficulties. However, nothing in neuroscience so far conflicts with the idea that a representation-as-percept is an input to something. It might be argued that standard causal principles only apply at the periphery of the system and not centrally, but it is unclear why or how. We have no reason to postulate an invisible envelope that divides an external or peripheral world from an inner ‘animate’ world (perhaps Fodor’s organism) with novel (i.e., supernatural) non-local properties, at any structural level. Neurobiology has shown that we can push the concept of ‘input’ as far in as interpretable empirical observation will allow, and well within the confines of the human body or brain. Pressure from an intervertebral disk on a lumbar nerve root gives pain in the foot. Cochlear implants give deaf people an experience of sound. Stimulation of cerebral cortex in the awake individual can evoke sensations and memories. The evidence indicates that sensory pathways, at all points up to that where a percept is experienced, are simply providing an input to the next stage, which often can be mimicked artefactually.

The work of Hubel and Wiesel (2005) and others has shown that detailed mechanisms of acquisition and collation of sensory data can be tracked far into the brain. Cells that respond to lines at particular angles, lines of limited length, or color contrasts can be demonstrated. It might be argued that the absence of precise analysis beyond this level could indicate that signals enter a ‘black box’ in which percepts are no longer associated with inputs, but rather with input-output relations. However, the simpler explanation is that beyond this level computation is so sophisticated that analysis requires very sophisticated experimental approaches. The more recent work of Quian Quiroga et al. (2005) showing that individual cortical cells respond to specific faces suggests that this is so.

In summary, despite speculations in other directions in some fields of study, the two assumptions of the neuron doctrine and the doctrine of percepts as based on inputs to perceiving entities appear to be worth retaining.

## Possible Domains for Representations as Percepts

Armed with this basic causal standpoint, it is possible to ask general questions about the location of the representations as percepts and the nature of the entities to which these are available. The starting premise is that at least one domain exists in a waking brain that supports an experience correlated with input from sense organs, contextualized by anticipations derived from kinesthetic monitoring, etc. We want to describe such a domain in dynamic physical terms. The prima facie case is that it will be a dynamic domain comprising part or all of one or more neurons, receiving inputs derived from all sensory modalities, and other internally generated signals, like names and concepts retrieved from memory (i.e., anything and everything we can experience), and capable of sending a sequence of outputs that can connect to all, or most, motor pathways. Conventional neuroscience indicates that the input will be of signals leading to patterns of depolarization of cell membrane. Since we are considering input this ought to be a pattern within dendrites (i.e., input projections).

It might be questioned that any single domain has inputs of all perceptual modalities and also concepts. However, our ability to mix raw sensory data and concepts in use of language indicates that somewhere in the brain signals with these disparate types of meaning are integrated – i.e., are co-inputs to some computational unit. Moreover, introspection indicates that human perceiving subjects experience them concurrently in a meaningful relationship and do so alongside the use of relevant language. Synchronization of signals may be important for optimal computation but as von der Malsburg (1981) pointed out when first suggesting that synchrony of signals was important, it can only be important *because it determines synchronized arrival* at some site of input.

I agree with Orpwood’s (2007) reasoning that percepts must be based on inputs that somehow are ‘interpreted’ on arrival at the perceiving domain and thereby have meaning to the perceiving subject. As this meaning belongs to the input itself, rather than any computational input–output relation, it seems that it too should be located at the site of input in dendrites. ‘Interpretation’ is not meant here in the sense that sensory signals encoding four legs, a bushy tail, pointed ears, and a toothy snout are converted to a signal meaning fox. That would imply at least one computation involving an input–output relation. The identification label ‘fox’ would be the input to the next domain along. Interpretation is used here to mean simply the correspondence of an input, (of electrical or chemical signals based on collation amongst sensory data and with data from memory) to a ‘percept’ that ‘is like something’ for, or has a meaning to, the receiving entity (in the above case legs, tail, ears, and snout). ‘Manifestation’ might be an alternative term, since it implies no additional physical interaction, but simply a correspondence between physical input and its meaning to the receiving entity.

Absence of a mechanism for this sense of interpretation may seem puzzling. However, immediate local correspondence between physical dynamics and meaningful experience seems to be something that, like Descartes, we have to take as brute fact. Ascribing it to processes prior to the point of input to the perceiving entity makes things no easier. There is no means by which to carry interpretation forward from previous events, since we have no evidence for anything other than the physical input itself being available to the receiving unit. Moreover, the idea of ‘carrying meaning forward’ generates an absurdity. Since the history of past events contributing to any causal interaction is immeasurably complex an immeasurably large number of ‘interpretations’ from earlier events should be carried forward in causal chains and that is not what we experience. Both the existence and the richness of the meanings inputs have to human perceiving subjects may be things for us to wonder at, but trying to delegate richness elsewhere is no solution.

It seems that representations as meaningful percepts ought to occur in neural dendrites.

## Representational Domains Cannot be Based on Traffic

A further consideration is helpful in narrowing down options for the domain of a percept. The content of a percept almost certainly has to be an interpretation of both signals associated with membrane excitation and ‘null signals’ corresponding to where membrane might have been excited but was not. Unless signals are interpreted in the context of all possible signals in a domain we lose what appears to be essential for a complex percept: encoding of information in patterns of inter-relation. A summation of all and only the black spots of a set of printed words can have only one meaning: black. (Or if black is coded null the sum of white areas just means white.) Moreover, it is indeterminate whether the spots included are on one page, or in a whole library. Only if both active and null signals and their relations are included do we have diverse meaning and bounded domains of meaning. In visual cortex, a ‘line’ of uniform color within a block of the same color is not interpreted as a line. The interpretation of ‘a line’ implies the absence of signals encoding similar color on either side of the line.

This means that the domain that supports a representation with meaning cannot be defined by a pattern of active signal traffic; it cannot be defined in terms of where signals are occurring. It must include null signals, so there must be some intrinsically defined structural domain within which signals and null signals are co-interpreted. The domain receiving signals interpreted as a percept cannot be an ‘active circuit’ in the sense of a set of pathways *currently* carrying signal traffic.

There is a distinction here between the processing units in a brain and in a computer. In a computer there are ‘gates’ in which electrical signals ‘open’ or ‘close’ connections between units, forming and breaking electrical circuits. The brain does not have gates in this sense. Connections remain unchanged, at least over periods of hours, regardless of traffic. The processing units are integrators, but not gates. Something akin to gating will occur during refractory periods and if input signals show differential synchronization in relation to refractory periods there may be triage, so that some active signals are ‘let through’ and others not. However, these signals will still operate in the context of null signals within the non-refractory time window.

## Localized Versus Distributed Representations

Representations-as-percepts, if only in a degraded form, survive damage to large areas of cerebral cortex. Damage to certain areas produces predictable defects, but does not appear to remove the capacity for some sort of perceptual experience, even if there is agnosia in the sense of not being aware that the percept is defective. The inference is that if the type of domain receiving representations as percepts is indeed cortical then there is no *single and local* domain. That leaves options of one very extended domain or multiple local domains.

The idea that a percept is an interpretation of the inputs to cells over a wide area of brain generates a range of problems, quite apart from the basic problem noted by James (1890/1983) that each cell’s input is separate. Many neurons are involved in ‘housekeeping,’ such as suppression of vision during saccades, or motor co-ordination. The inputs to such cells do not appear to figure in percepts, which reflect the input to a select cell population involved in a field of attention. It is unclear, in a distributed model, why the inputs to certain cells and not others should figure in a reportable percept. Nor is it clear why we should perceive a single ‘copy’ of sensory data if cells over a wide area contribute, since most if not all signals arising from cellular activity in sensory pathways are sent as inputs to many cells through widely ramifying axonal branches. When we see a red tomato early signals referring to a red tomato are sent to 1000s of cells further forward in the brain. Why should we consider these thousands of ‘copies’ a single representation? If a company sends out 1000 Christmas cards, each with a photo of head office in the snow, do we consider this ‘one representation’ of head office?

These and related concerns may have motivated the proposal by Pribram (1991) that the cortex carries information somewhat in the manner of a hologram, in which every part of a spatial array carries a copy of the entire pattern of information being handled. Although often thought of as a model of distributed representation, the holographic model provides a means for having very many ‘copies’ of a pattern at many sites rather than a single copy available to one extended site. A simpler and neurologically reasonable version of the idea is just that sensory data are sent to many locations in the cortex and each of these has the potential to interpret its input as percept. This would seem to be in keeping with the experiments of Quian Quiroga et al. (2005) in which visual sense data often gave rise to excitation in many sampled cortical cells. In some cases cells were highly restricted in their responses to images, but others are more promiscuous. At least there is little doubt that sensory stimuli lead to signals being sent widely to many cells.

In summary, although the discussion so far might suggest that the question of what domain supports a perceptual representation is just what it must always have been – which cell or cells – it may need a subtler formulation. How many of which sort of neuron have a perceptual representation encoded in their input(s) and do they constitute one domain of one representation of this type at any one time or are there multiple domains, with multiple representations based on the same sensory data? It is important to note that the latter should not be expected to evoke a *sense* of multiplicity (of the perception of being one of many subjects) since multiplicity would not itself be encoded, represented or, therefore, perceived by anything, being a fact about parallel reception events, not a property of the receiving unit, or the content of its input.

At this point the reader may sense that the concept of representation is too confusing to be useful, and there is a case for that position! I would argue, however, that if some historic confusions in the literature are unpacked it is possible to restore the usefulness of the idea, with some riders that add significant explanatory power.

## Pontifical, Grandmother and Cardinal Cells

The simplest hypothesis for the domain of a percept, now universally taken as a null hypothesis, is that of a single pontifical cell, as discussed by James (1890/1983) and dating back at least to ideas raised by Leibniz (Woolhouse and Franks, 1998), writing shortly after cells were first observed. This form of pontifical cell is a single cell that supports all ‘my’ representational percepts of all sensory inputs. It is the ‘me’ cell. Other cells act as conduits to and from this central cell, collating inputs and delegating outputs. James considers that they might also support ‘percepts,’ but of a meaner sort than those I report as ‘mine.’ (He includes the point that none of these percepts need involve any sense of multiplicity or presence of others.) The attraction of this idea is that the cell is the brain’s integrating unit, with an intrinsically delimited input domain, and the contents of human experience appear to be integrated and delimited. However, the idea that just one neuron should have this specialized function is implausible on a range of grounds and, as indicated above, the argument that experience *seems* ‘single’ is immaterial, since there would be no reason for there to be representation (and thus perception) of multiplicity, or a sense of ‘other copies,’ within each of multiple representations.

It is useful to raise here a potential confusion in terminology between sites of representation and sites of recognition. Sherrington (1940) invoked a concept of a *quite different* sort of ’pontifical’ cell to explain recognition. Sensory data relating to an object such as a dog enters through many 1000s of receptors. Recognition would appear to require sequential stages of discrimination, each leading to a reduced number of possible interpretations. This might be expected to form of a ‘pyramid’ with fewer cells at each stage until the input finally converged on one cell responsible of recognizing dogs. There would be a pontifical cell for a dog, another for a cat and another for grandmother.

A key point here is that we have no reason to think that only the cell with the job of recognizing dogs will receive input signals encoding doggy features. If 100 cells each recognized a different mammal we would not expect the presence of a dog to lead to input to only one of these. We would expect all the cells to receive signals encoding doggy features but only one (or some) to fire. It could be argued that synapses receiving signals encoding long snouts will atrophy on koala-recognizing cells but at least to be able to learn to recognize new animals we have to assume that cells with catholic inputs exist.

**FIGURE 1:**
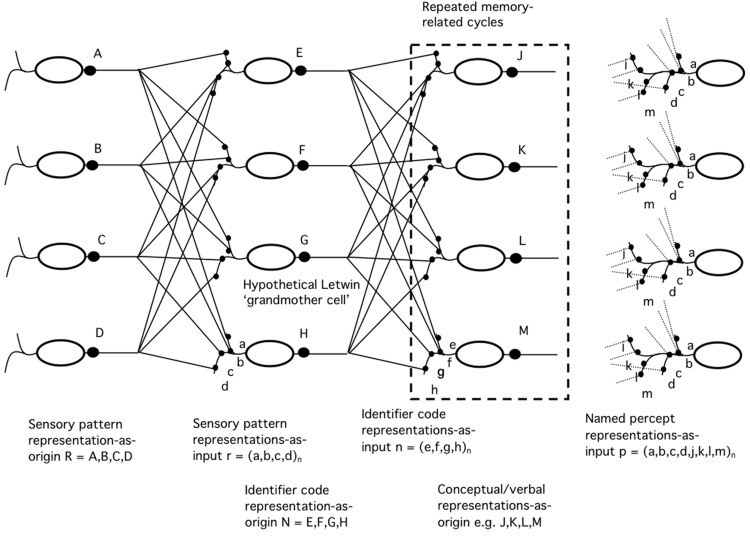
**Simplified schema of successive representations-as-origin and representations-as-input, starting with sensory patterns, followed by recognition with encoding as identifiers that allow recall from memory of concepts and finally the generation of percepts including both sensory patterns and conceptual/naming features**.

Thus if a representation is based on an input pattern we do not expect sites of representation and recognition to be commensurate. This emphasizes the need to consider a causal chain as potentially involving many levels of representation with multiplicity at each level (**Figure 1**). It highlights the fact that a representation is always a step in a causal chain and is thus always a representation *to* a domain at a particular point in that chain. Thus a pattern of data, perhaps encoding legs, fur and muzzle, would represent a dog *to* a ‘dog-pontifical cell’ as well as to a lot of other cells, untuned, or tuned to other creatures. In turn the firing of the dog-pontifical cell *and not* its neighbors would denote ‘dog’ to the rest of the brain. The two types of representation would be quite different. Moreover, intuition tells us that whatever domain has a percept of a dog of the sort normally discussed it must have an input encoding both the key features of a dog – legs, fur, etc. – *and* the sense of these being part of a dog, apparently putting the relevant domain downstream of the site of dog-recognition with additional parallel input encoding the original upstream context-dependent sensory data.

Empirical studies indicate that recognition does not use a pyramidal system with fewer and fewer cells at each stage (Barlow, 1972). Sequential stages involve as many, if not more, cells as at the beginning – as implied by the above discussion. Recognition is signaled by the firing of one or a few cells in the context of non-firing of many more cells. At all stages representations are thus widespread, but it needs to be established whether this is because individual representations are extended or because of multiplicity.

This issue is relevant to Barlow’s (1972) classic Perception paper. Barlow takes as his object grandmother, following Letvin (Gross, 2002) and discusses the plausibility of a ‘grandmother cell’ in the sense of a single cell that fires with 100% sensitivity and specificity for grandmother. This bears a relation to Sherrington’s (1940) pontifical cell but not to that of Leibniz or James. Barlow suggested that grandmother was probably not important enough to have her own cell and that, more likely, grandmother would be encoded by the activity of perhaps a thousand ‘cardinal’ cells, each representing an aspect of grandmother such as a mouth or nose, any of which might presumably contribute to encoding other faces in other combinations. These elements of the percept are then seen as combining rather in *the way words combine in a sentence* (an analogy also used by Marr, 1982). Note that Barlow is not proposing a redundancy-for-safety strategy with information distributed in a ‘holographic’ way to several cells, each with a sensitivity and specificity of less than 100%. He is giving each cell a separate and specific job.

The odd thing here is that Barlow appears to be describing the activity of cells upstream of a site of recognition of grandmother. If each cell is responding to signals which together encode a feature not entirely specific and sensitive for grandmother then grandmother can only be recognized, and social responses activated, by a downstream group of cells receiving inputs from these thousand cells, *some of which downstream cells will fire and some not*. It would be these downstream cells whose inputs would encode all grandmother’s features and it would therefore be their domains that we could (perhaps) expect to support a ‘percept’ of granny in the sense of manifestation of all of grandmother’s features, whether or not they fired. And it would be the pattern of firing and non-firing of these latter cells that would ‘represent’ (in the denoting sense) to domains in the rest of the brain the presence, but not the pattern of features, of this individual. Whether or not within this latter group of cells there are cells with 100% sensitivity and specificity for grandmother is a different issue that need not bear on the search for the domains supporting the representations known as percepts.

More recently, Quian Quiroga and Kreiman (2010), has discussed the interpretation of experiments on individual cell responses to faces (Quian Quiroga et al., 2005) in relation to the grandmother cell concept. In this case the grandmother cell is rejected on redundancy grounds. While emphasizing the complexity of the grandmother cell concept, discussion seems to bypass the crucial issue of the distinction between the site of experience of a pattern such as a face and the site of recognition of such a pattern. Nevertheless, it seems to support the idea that inputs carrying information about a pattern such as a face will be received by not one, but many cellular computational units.

The above discussion emphasizes a number of issues relating to this crucial question. It seems that representations (in the broadest sense) of a given referent in the brain must be multiple and diverse. At each level many cells will be involved in representing. Representation and recognition are not likely to be commensurate. So far the discussion has been in terms of individual cells despite the general assumption in the literature that representations in brains each involve many cells. The grounds for such an assumption need be revisited in the light of the preceding arguments.

## A Return to the Neuron Doctrine

As already noted, to be useful, the concept of representation-as-percept, has to imply a step in a causal chain with content encoded in the input to some domain. It is also difficult to see how a representation can have a meaning, or interpretation, to a domain, unless its content is encoded in the co-temporal input of a pattern of active signals and null signals to the domain. Representations like this do not occur in computers. Stored data in a computer can represent something meaningful to a human user accessing it via a screen but no representation based on a pattern of co-temporal input occurs *to* anything within the machine beyond the four trivial input options for an electronic gate of on/on, on/off, off/on and off/off. Moreover, we do not require that anything in a computer interprets, or attributes meaning to, input signals. It might be argued that a sequence of signals passing through a gate might constitute a representation. However, since each signal contributes to a separate computation this is problematic. The sequence of incoming signals is not subjected as a whole to a computation, other than as arbitrarily defined by a programmer. Within the machine any temporal ‘chunking’ of serial signals into ‘representations’ adds nothing to the causal account and at the gate in question no chunking should be apparent.

Within brains there *are* units that receive complex patterns co-temporally: neurons. Moreover, they are the only units that receive patterns relevant to percepts as far as we know. Barlow’s 1000 cardinal cells are not a unit receiving a pattern of features of grandmother. Each has a separate input encoding one feature. For all 1000 features to contribute co-temporally to a representation 1000 cardinal cells must send all 1000 active or null signals to converge on at least one downstream neuron, which is within the range of neuronal inputs. The neuron doctrine, as was probably evident to Leibniz, entails the simple but surprising conclusion that representations qua percepts in brains can only be in individual neurons (Edwards, 2005; Sevush, 2006, 2016). There may be very large numbers of such representations, all encoding the same sensory data, distributed over a wide area, but each percept must be tied to the receiving unit that is the neuron.

This conclusion immediately resolves the paradox of localization and distribution of representation in the brain, since it implies that *local* representations can be present over a *widely distributed area*. This situation is familiar in the distribution of a newspaper, which is widespread but can only convey news if all the words of a news story are present in each copy read by an individual. To suggest that a single perceptual representation could be available to several cells is equivalent to saying that news can be understood by a group of people each of which receives one word from the paper.

The conclusion also resolves the question of precisely where in the brain are the representations that determine our actions. The answer is that they may be all over the brain. Even the question of where in the brain are the representations that determine considered verbalized behavior may have the same answer, although it seems reasonable to attach some special significance to representations in cells with multimodal inputs that would allow both the visual and auditory features and the concept of a dog to contribute to a ‘percept’ of a dog.

Putting representations in individual cells may appear implausible. However, it is unclear why a representation in a single cell should be more implausible than one involving many cells. The implausibility may be more salient simply because any proposal for a specific location for such representations brings into focus our lack of understanding of the rules of interpretation. This may be no bad thing. Ironically, the charge of implausibility tends to come from those who argue for functional rather than structural analysis and yet the conclusion is based on the ‘functional’ property of having input (and capacity) rather than structure. The conclusion might be branded over-reductive but one of its key features is that it makes explicit the boundary between reductive analysis and the non-reductive relation of ‘interpretation,’ rather than invoking an ill-defined internal no-man’s-land where both are claimed to apply at different ‘levels.’

Another attraction of the idea that representations are *to* the single computational (rule based input–output) units that are neurons is that it implies that the brain does not perform single operations on ‘atomic’ (structureless) symbols, but rather it performs operations on ‘molecular’ representations. That is to say that the basic data units that the brain operates on are irreducibly complex, with many degrees of freedom. This begins to address the puzzle of how the manipulation of symbols can be associated with an experience of complex patterns that reflect the complexity of their referents. It also provides a reason why, as appears to be increasingly recognized, syntax and semantics cannot be totally dissociated when considering meaning (Hinzen, 2006).

## Multiple Representations of Multiple Types

The concept of multiplicity of representations of sensory data in the brain should not be unexpected if we consider the parallel and hierarchical nature of computation. There may be a lingering presumption that representations, qua percepts, ought to be single – belonging to a single ‘me,’ but this is not logically required. There is also a lingering discomfort with the idea that our actions may be guided by representations distinct from those we report as our percepts. Perhaps the best known ‘redundancy’ of representations is that implied by the dual path hypothesis for visual perception of Goodale and Milner (1992). The dissociation of percept and action described by Króliczak et al. (2006) for the hollow face illusion, presents the counterintuitive idea that the brain builds more than one spatial representation, which might seem redundant or extravagant in use of resources. This has the interesting implication that we consider the building of spatial representations qua percepts labor-intensive.

**Figure 1** illustrates an approach to representation in the brain that suggests that this concern may be misplaced. It makes explicit the idea that ‘representation’ has two different meanings. One sense of representation (R) is an instance of a pattern, as in a picture or map, that acts as *origin* for a representation in the other sense (r) of an instance of representing *to* something via its *input*. At every stage of neural computation we can expect a representation-as-input to lead to an output that can act as representation-as-origin for the next stage. At every stage banks of cells will be involved but whereas such a bank of cells will hold a single representation-as-origin it will hold as many representations-as-inputs as there are cells in the bank. Perhaps surprisingly, although building a representation-as-origin is likely to be labor-intensive, much larger numbers of representations-as-inputs, which we could expect to correspond to percepts, would appear to come free of charge.

We are used to the idea that the nervous system generates motor output from sensory input at several levels of complexity. There are spinal reflexes, brainstem reflexes, automatic but co-ordinated responses involving cerebellum, routine purposive actions and deliberated actions. All of these can be expected to be associated with different levels of representation-as-origin and representations-as-input so we should not be surprised by the idea of multiple spatial representations even in terms of representations-as-origin. Perhaps more interestingly, as indicated on the right side of **Figure 1**, hierarchies of representation-as-origin give the opportunity for representations-as-input downstream to ‘pick-‘n’-mix’ inputs from more than one level of this hierarchy. Thus there is nothing very surprising about the idea that the representations that guide our rapid actions appear to overlap in content in most but not all situations with those that form the basis of our percepts.

## Conclusion

Mainstream neuroscience prides itself in being rigorously physicalist, in the sense of adhering to the basic precepts of natural science and general principles of causality. A consideration of representations in such a rigorous causal framework leads to the conclusion that all representations in the brain, including those that may form the basis of percepts, must ultimately be considered in terms of how they are cashed out in the inputs to individual neurons. These representations as inputs will occur at multiple levels of sensory processing and will be multiple at all levels, including levels associated with pattern recognition, denotation and reportable percepts. Such a model is counterintuitive but resolves certain important problems relating to the distributed nature of representation and may provide clues to the basis of meaning and language.

## Author Contributions

The author confirms being the sole contributor of this work and approved it for publication.

## Conflict of Interest Statement

The author declares that the research was conducted in the absence of any commercial or financial relationships that could be construed as a potential conflict of interest.
